# Bioequivalence assessment of two Ticagrelor formulations under fasting condition in healthy Pakistani subjects

**DOI:** 10.12669/pjms.39.6.8203

**Published:** 2023

**Authors:** Naghma Hashmi, Masood Jawaid, Muhammad Raza Shah

**Affiliations:** 1 Naghma Hashmi Center for Bioequivalence Studies and Clinical Research, Dr. Panjwani Center for Molecular Medicine and Drug Research International, Center for Chemical and Biological Sciences, University of Karachi, Karachi, Pakistan; 2 Masood Jawaid Director Medical Affairs, PharmEvo Private Limited, 402, Business Avenue, Block-6, P.E.C.H.S., Shahrah-e-Faisal, Karachi, Pakistan; 3 Muhammad Raza Shah Center for Bioequivalence Studies and Clinical Research, Dr. Panjwani Center for Molecular Medicine and Drug Research International, Center for Chemical and Biological Sciences, University of Karachi, Karachi, Pakistan

**Keywords:** Bioequivalence, Ticagrelor, Bioavailability, Pharmacokinetic

## Abstract

**Objective:**

To investigate the Bioequivalence of Anplag® 90mg (Ticagrelor) tablet and Brilinta® 90 mg (Ticagrelor) tablet under fasting conditions in healthy Pakistani subjects.

**Method:**

This was an open-label, cross-over, randomized, single-dose, two-period, single-center Bioequivalence Study conducted at Center of Bioequivalence Studies and Clinical Research (CBSCR), ICCBS, University of Karachi, Karachi, Pakistan from September 2020 to January 2021. This was an open-label, randomized, single-dose, two-period, cross-over Bioequivalence Study. After randomization, a single dose of Ticagrelor 90mg tablet (test or reference drug) were administered orally in 1:1 ratio to each subject under fasting conditions. Seven days washout period was kept between the two periods in order to avoid carry over. Blood samples were then taken up to 48th hours post-dose. Point estimates and 90% confidence intervals (CI) for the ratio of the log-transformed values were calculated. Bioequivalence assessment of both, the reference and the test drugs were based on the primary Pharmacokinetic PK metrics including peak maximum concentration (Cmax), area under the curve (AUC) from zero to last quantifiable concentration (AUClast), and AUC from zero to infinity (AUCtotal) after log-transformation of data with ANOVA. In this bioequivalence study, the primary pharmacokinetic parameters were assessed for both Ticagrelor and its Active Metabolite (AR-C124910XX). Safety endpoints were evaluated by monitoring adverse events (AEs).

**Results:**

The 90% Confidence Intervals (CIs) of the Geometric Mean Ratio for primary PK parameters including C*max*, AUC*last*, and AUC*total* all were within the accepted bioequivalence range of 80%- 125%. In the current study, no serious adverse events were reported.

**Conclusion:**

Our results showed that the two tested formulations of Ticagrelor tablets were bioequivalent and well tolerated.

***Trial Registration:*** ClinicalTrials.gov Identifier: NCT04941196

## INTRODUCTION

Ticagrelor is an oral, reversible, antiplatelet agent which has been established with the aim of preventing atherothrombotic events in patients diagnosed with acute coronary syndrome (ACS).^1^ Acute coronary syndrome (ACS) refers to a constellation of clinical syndromes resulting from thrombus formation leading to myocardial ischemia. It is a serious health problem with high morbidity and mortality.^2^ Recommended treatment options for ACS include dual antiplatelet therapy (DAPT) in addition to acute management. Studies have shown the beneficial effects of using Aspirin plus P2Y12 inhibitors (Clopidogrel, Prasugrel, or Ticagrelor).

Ticagrelor is the first direct-acting P2Y12 inhibitor, having a rapid onset of action. It was recommended by the United States Food and Drug Administration (FDA) in 2011 and European Medical Commission in 2010 to be used with low-dose aspirin in patients with acute coronary syndromes so as to minimize the risk of stroke and myocardial infarction.^3^

**Fig.1:**
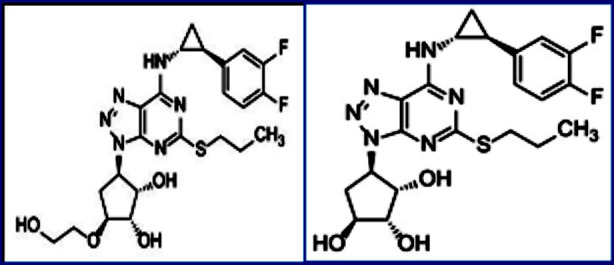
Structures of Ticagrelor (1) and its active metabolite M8 (2).

Ticagrelor belongs to a new chemical class cyclopentyltriazolopyrimidines, with a unique mechanism of action that results in a direct reversible competitive inhibition of the P2Y12 receptor. Ticagrelor is an adenosine triphosphate analog and reversible P2Y12 Purino-receptor antagonist that inhibits ADP-mediated platelet aggregation. Ticagrelor act as a Phenylalanine Hydroxylase Activator, Cytochrome P450 3A4 Inhibitor, Cytochrome P450 3A5 Inhibitor, P2Y12 Receptor Antagonist, and P-Glycoprotein Inhibitor. Ticagrelor and its major metabolite (Fig.1) interact with the platelet P2Y12 ADP- receptor reversibly resulting in reduced signal transduction and platelet activation.^4^ Ticagrelor and its active metabolites are excreted mainly by bile and a small amount by the kidneys.^5^

Previous studies have shown higher efficacy with comparable safety of Ticagrelor than clopidogrel, therefore Ticagrelor is now recommended as the first-line treatment for ACS patients in Dual Antiplatelet Therapy (DAPT).^6^,^7^ Few other studies have also shown that compared to dual antiplatelet therapy, it is not associated with a higher risk of cerebrovascular events.^8^,^9^ Although the branded drug Ticagrelor is available for clinical use for many years, its use is limited for patients with ACS in developing countries owing to high cost. Therefore, a generic version of Ticagrelor was evaluated in this study for bioequivalence as part of the approval process for marketing authorization of this product which will help in improving the access to a vast number of patients.

The aim of this study was to compare the bioequivalence, safety, and tolerability of the Ticagrelor test drug Anplag® 90mg manufactured by PharmEvo Private Limited, with the originator drug Brilinta® 90mg, manufactured by AstraZeneca Pharmaceuticals in healthy Pakistani volunteers under fasting condition to comply with the FDA and other international regulations for drug marketing.

## METHODS

### Formulations

The Ticagrelor test drug Anplag® 90mg manufactured by PharmEvo Private Limited., and the reference drug Brilinta® 90mg manufactured by AstraZeneca Pharmaceuticals, Co., Ltd.

### Study site and Subjects

The study was conducted at the Center of Bioequivalence Studies and Clinical Research (CBSCR), ICCBS, University of Karachi, Karachi, Pakistan from September 2020 to January 2021.

### Inclusion & Exclusion Criteria

Participants were selected based on the clinical assessment & clinical laboratory examination. Healthy human subjects fulfilling the inclusion and exclusion criteria were enrolled for participation in the final study. Eligibility criteria for age were between 18 to 55 years and 18.5 to 30 kg/m2 (both inclusive) for BMI. Healthy subjects were selected as governed by routine physical examination, including vital sign monitoring (i.e., blood pressure, heart rate and temperature), 12 lead ECG, and laboratory analysis (i.e., hematology, blood biochemistry, serology and urinalysis). Subjects were free from any epidemic or contagious diseases. Heavy smokers, and Subjects with history of any illness that, in the opinion of investigator might confound the result of the study and post additional risk in administrating Ticagrelor to the subject, were excluded from the participation. After the completion of the screening process, 31 healthy subjects reported on the check-in day of Period-I and were assigned study-specific codes. This sample size was selected based on the intra subject variability for Ticagrelor and AR-C124910XX for AUC and Cmax assumed to be ≤24%^10^ a sample size of approximately 30 volunteers were required to show bioequivalence with 90% power and an α-level of 0.05.

### Ethical Approval

The study followed the Declaration of Helsinki, the International Council for Harmonization (ICH)–Good Clinical Practice (GCP), and other recommended guidelines for the conduct of bioequivalence (BE) studies. The study was approved by the Independent Ethics Committee University of Karachi, Karachi. Pakistan, National Bioethics Committee (NBC) Islamabad, Pakistan (Ref: No: 4-87/NBC-448/19/1730) and Drug Regulatory Authority of Pakistan (DRAP).

Informed consent was obtained from the subjects before enrollment and prior to any screening procedure. Volunteers were briefed on the objectives, procedures, and potential risks of participation in the study. After the subjects qualified for the screening procedure and inclusion/exclusion criteria, the participants signed the informed consent forms (ICF) for participation before their partaking in the clinical phase.

### Study Design

This was a single center, single dose, two-period, randomized, open-label, cross over Bioequivalence Study of Anplag® 90mg (Ticagrelor) tablet & Brilinta® 90 mg (Ticagrelor) tablet in healthy adult Pakistani subjects under fasting condition. After overnight fasting of approximately 10 hours, the Test drug (Anplag®) and Reference drug (Brilinta®) tablet 90mg single dose was administered to subjects with 240 ml of water according to the randomization list as per scheduled time. Subjects received one single dose per treatment period of test or reference drug separated by a wash-out period of seven days. Subjects remain seated for four hours after drug administration, while no exercise or strenuous physical activities were permitted during this period. Subjects received standardized meals which were provided at the same time in each period of the study. During each study period, the subjects were allowed to consume water as desired except for one hour before and after the dose.

Blood samples (4 mL of venous blood) were collected at pre-dosing (0.0 hour) and at 0.5, 1.0, 1.5, 2.0, 2.5, 3.0, 3.5, 4.0, 5.0, 6.0, 8.0, 10, 12, 14, 24, 36 and 48 hours after drug administration into a vacuum anticoagulation tube which contained Heparin lithium. After collection, the blood samples were centrifuged at approximately 4000 rpm for five minutes at 4±1^o^C. The plasma samples were then aliquoted into three parts, and collected samples were stored within one hour at -20°C and transferred after 48 hours to -80°C for storage until analysis.

According to FDA recommendations (Guidance document on Ticagrelor Bioequivalence), the analytes to be measured were Ticagrelor and its active metabolite, AR-C124910XX in plasma, while the bioequivalence will be established only on the parent drug (Ticagrelor).^11^ Hence to consider the FDA recommendation, plasma samples of 30 volunteers were quantified using a validated High-Performance Liquid Chromatography (HPLC) method. An isocratic HPLC method was performed on a C8 column (150cm x 4.6mm., 5µm) with the mobile phase system consisting of Methanol: 8mM NH_4_HCO_3_ buffer-A pH 3.20 (59:41 v/v) and a column temperature were maintained at 60°C with an injection volume of 85µl. Each volunteer sample were analyze in single analytical run.

### Safety Assessments

All subjects were monitored clinically for health status during both periods of study and vital signs were measured at the time of check-in, before dosing (0.0 hour), and at 2, 4, 8, 12, 24, 36, and 48 hours after drug administration. Evaluation of safety endpoints was done by performing physical examinations including pulse, blood pressure, and body temperature measurements, followed by clinical laboratory tests including serum biochemistry, urinalysis, serology and a PT/INR test were also checked to see bleeding or clotting disorders; plus, electrocardiogram (ECG) for adverse events (AEs). Subjects were tested for COVID-19 (through a Rapid COVID-19 antibody testing kit) at the time of the screening phase and Check-in, in each study period.

### Pharmacokinetic and Statistical analysis

All Pharmacokinetic and statistical calculations were carried out with the validated commercial pack KINETIKA Software Version 5.1 (Thermo Scientific), using non-compartmental modeling. Descriptive analysis was used to assess the demographic characteristics, including age, height (cm), weight (kg), and BMI (kg/m2). C*max* and T*max* were determined directly from the observed plasma concentration-time data. AUC was calculated using a trapezoidal rule. Analysis of variance was conducted using formulation, sequence, and period as fixed effects. Point estimates and 90% confidence intervals (CI) for the ratio of the log-transformed values were calculated. ANOVA was calculated for the log-transformed values of C*max*, AUC*total*, AUC*last*, T*max*, and t*_1/2_* of Ticagrelor and metabolite. If the two-sided 90 % confidence intervals for the test to reference ratio of the means were within 80.00 – 125.00% for the ln transformed data of primary pharmacokinetic variables including C*max*, AUC*total*, and AUC*last* of Ticagrelor the average bioequivalence of the drugs was concluded.

## RESULTS

### Subjects Disposition

The ages of all the 31 subjects (all males as no female reported the clinical site) were in the range of 18.1- 36.1 years (acceptance range: 18 to 55 years) while their BMI range was between 18.9- 29.7 kg/m2 (acceptance range: 18.5 to 30 kg/m2) (Table-I). A total of 114 subjects visited for screening, out of which 93 were checked in for screening process as 14 subjects were found positive for COVID-19 IgG antibodies while 07 subjects did not proceed for check-in after the COVID-19 monitoring test. Thirty-one healthy subjects were enrolled for the study, while one subject was disqualified based on pre-dose safety assessment and hence thirty subjects were finally dosed and completed the study.

**Table-I:** Baseline demographic characteristics.

	Mean (SD)	Range
Gender	Male	
Ethnicity	Asian (Pakistani)	
Age, (Years, month)	25.5± 4.4	18.1-36.1
Height (cms)	165.7±6.3	154-178
Weight (Kg)	63.7±8.1	50-81
BMI (Kg/m^2^)	23.3±3	18.9-29.7

### Safety Assessments

Vital signs were measured as a safety assessment at the time of, check-in before dosing (0.0 hour), and at 2, 4, 8, 12, 24, 36, and 48 hours after drug administration. A total of six adverse events (AEs) were documented throughout the entire study duration which were mild and moderate in nature. These events included headache, epigastric fullness (vomiting), hypertension, constipation, and diarrhea. Out of these, three AEs were reported by participants who received the reference drug, while the remaining AEs were associated with the test drug. One study subject, however, experienced high blood pressure before drug administration (pre-treatment adverse event) and dropped out of the study. All the adverse events were not serious and thus Anplag® and Brilinta® were considered to be safe in the tested population. Both formulations of Ticagrelor were tolerated well by all study participants.

### Bio-analysis

Plasma samples of 30 volunteers were quantified using a validated HPLC method with a linear calibration range of 30-1360 ng/ml for Ticagrelor (TIC) and 9-408 ng/ml for active metabolite (AR-C124910XX) of Ticagrelor. The correlation coefficient (r) was always greater than 0.99 during the whole validation and analysis phase.

**Fig.2:**
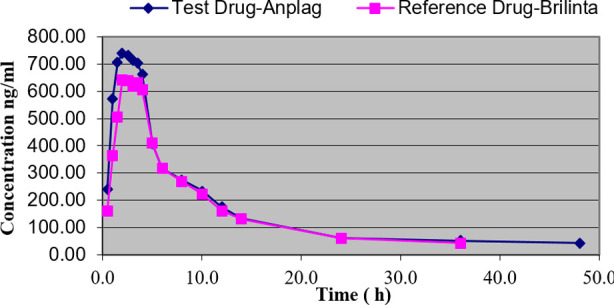
Mean plasma concentration–time curves of Ticagrelor under fasting condition-Pharmacokinetic Analysis Concentration Set (PKCS).

The mean plasma Ticagrelor concentration versus time profile at each time point for all the volunteers was obtained with Anplag® & Brilinta® 90mg (Ticagrelor) tablet. Overall, the plasma profile in Fig.2 showed that the peak plasma Ticagrelor concentrations were attained at approximately two hours.

### Pharmacokinetic and Bioequivalence Assessment

Based on actual blood-sampling time points, pharmacokinetic (PK) parameters were calculated from the plasma concentration-time data for both products. These parameters include C*max*, the area under the concentration-time curve from 0 to the last measurement (AUC*last*) and from 0 to infinity (AUC*total*), Time to reach maximum concentration (T*max*) and half-life i.e., the time required for Ticagrelor and its active metabolite concentration to reduce to half (t1/2).

The analysis of pharmacokinetic parameters for bioequivalence study was performed on the pharmacokinetic (PK) samples of 30 volunteers, however; bioequivalence was concluded on 29 volunteers as one volunteer showed maximum drug concentrations (C*max*) at eight hours for reference drug (Brilinta®) in Period-II of the study, which is far beyond the range (1-5 hours.) thus was considered as an outlier. The pharmacokinetic parameter values obtained in this study were presented as geometric mean (± SD) for both Test and Reference formulations (n=29). All major PK parameters lie within the acceptable limits range of 80.00-125.00 % for AUC*last*, AUC*total*, C*max*, and t*_1/2_* except T*max* for Ticagrelor. T*max* is a secondary Pharmacokinetic variable and according to the European Medicines Agency guidelines on “the investigation of bioequivalence”, the parameters to demonstrate bioequivalence are C*max* (Peak concentration), and the Area under the concentration-time curve (AUC) and hence, statistical evaluation of T*max* is not required.

Individual and mean concentrations, individual and mean pharmacokinetic parameters, and geometric means and ratios of means for AUC*last*, AUC*total* and C*max*, of the Ticagrelor active metabolite (AR-C124910XX) have also been reported. The pharmacokinetic properties of Ticagrelor and its active metabolite from both products are summarized in Table- II.

**Table-II:** Geometric means, Percentage ratio of Geometric means and 90% Confidence Interval results from ANOVA.

TICAGRELOR (N=29)				

Parameter	Geometric Means		% Ratio of Geometric Means	90% Confidence interval

	Test	Reference		
AUC*last* (h×ng/ml)	6061.85	5412.39	112.33	106.63 – 118.35
AUC*total* (h×ng/ml)	6610.63	6071.84	109.14	103.66 – 114.92
C*max* (ng/ml)	1000.9	860.23	116.52	108.87 – 124.72

*TICAGRELOR METABOLITE (AR-C124910XX) (N=29)*				

AUC*last* (h×ng/ml)	1452.71	1306.8	111.17	-
AUC*total* (h×ng/ml)	1687.92	1575.59	107.20	-
C*max* (ng/ml)	172.593	144.498	119.27	-

## DISCUSSION

Currently, Ticagrelor is recommended over clopidogrel (an older antiplatelet drug) for the prevention of thrombotic events in patients with acute coronary syndromes. Clopidogrel is a prodrug which is absorbed through the intestine and activated in the liver while Ticagrelor is not a prodrug, therefore it shows rapid antiplatelet activity without any prior metabolic activation^11^

Secondly, in up to one third of patients receiving clopidogrel inadequate antiplatelet effects are observed.^12^-^14^ This variation is mainly due to the presence of the polymorphisms in genes such as CYP2C19 and ABCB1, whereas the CYP2C19 genotype don’t have any effect on the antiplatelet activity of ticagrelor.^15^ Therefore, a cost-effective brand alternative in the form of generic Ticagrelor is the need of the day.

The main objective of bioequivalence studies is to assure the bioavailability and safety of generic formulations; hence the present study was conducted to compare the bioavailability and safety profiles between the generic Ticagrelor and brand drug in healthy adult Pakistani subjects under fasting conditions. This is the first bioequivalence study of Ticagrelor, reported on Pakistani population.

The results of the in-vitro study including assay showed 99.4% for reference formulation and 100.8 % for the test, similarly the dissolution tests were found to be 101% and 99% for test and reference drugs respectively. Although the differences found in the potency and dissolution test were small indicating that probably the products would have a similar performance in-vivo. The comparative Dissolution Profiling (CDP) data of both the formulations were also comparable.

The bioequivalence acceptance criteria were applied to Ticagrelor pharmacokinetic parameters and the study demonstrated that the primary PK parameters, including C*max*, AUC*last*, AUC*total*, were similar in the two studied formulations of Ticagrelor. According to the FDA Guidance on Ticagrelor, Ticagrelor active metabolite (AR-C124910XX) data should also be reported as supportive evidence of comparable therapeutic outcome.^11^ All volunteers were male, as no female volunteers reported the site despite the advertisement for volunteers’ recruitment addressing both male and female. This gender specific trial do not affect the PK parameters as it was a comparison study and both test and reference formulations had the same impact.

### Adverse Effects

A total of six AEs (T=3, R=3) were recorded and out of which five were thought to be drug related while only one (hypertension) were not related to drug. All reported adverse events were mild and moderate and were resolved during the study without any special intervention. Headache and Abdominal pain were categorized as mild while rest of the AE including vomiting, constipation and hypertension were moderate in nature. There was no major difference in the AEs between the test and reference drug groups. Therefore, both formulations of Ticagrelor were well tolerated by healthy Pakistani subjects.

### Limitations

Participation of the female volunteers might overcome the gender biasness.

## CONCLUSION

The present study concludes that the tested Anplag® 90mg (Ticagrelor) tablet by PharmEvo Private Limited is bioequivalent to the reference Brilinta® 90mg (Ticagrelor) tablet manufactured by AstraZeneca LP. The two formulations were well tolerated indicating that the generic Ticagrelor can be interchangeable with the innovator drug.

### Author Contribution:

**NH:** The first draft of the manuscript was written, analysis & interpretation of data were performed.

All authors contributed to the study conception & design, read and approved the final manuscript.

## References

[1] Deeks ED (2011). Ticagrelor:a review of its use in the management of acute coronary syndromes. Drugs.

[2] Virani SS, Alonso A, Benjamin EJ, Bittencourt MS, Callaway CW, Carson AP (2020). Heart disease and stroke statistics-2020 update:a report from the American Heart Association. Circulation.

[3] Tersalvi G, Biasco L, Cioffi GM, Pedrazzini G (2020). Acute coronary syndrome, antiplatelet therapy, and bleeding:a clinical perspective. J Clin Med.

[4] Kubisa MJ, Jezewski MP, Gasecka A, Siller-Matula JM, Postula M (2018). Ticagrelor –toward more efficient platelet inhibition and beyond. Ther Clin Risk Manag.

[5] Rosa GM, Bianco D, Valbusa A, Massobrio L, Chiarella F, Brunelli C (2016). Pharmacokinetics and pharmacodynamics of Ticagrelor in the treatment of cardiac ischemia. Expert Opin Drug Metab Toxicol.

[6] Amsterdam EA, Wenger NK, Brindis RG, Casey Jr DE, Ganiats TG, HolmesJr DR (2014). AHA/ACC Guideline for the management of patients with non-ST-elevation acute coronary syndromes:a report of the American College of Cardiology/American Heart Association Task Force on Practice Guidelines. J Am Coll Cardiol.

[7] Collet JP, Thiele H (2020). The 'Ten Commandments'for the 2020 ESC guidelines for the management of acute coronary syndromes in patients presenting without persistent ST-segment elevation. Eur Heart J.

[8] Hiasa Y, Teng R, Emanuelsson H (2014). Pharmacodynamics, pharmacokinetics and safety of Ticagrelor in Asian patients with stable coronary artery disease. Cardiovasc Interv Ther.

[9] Li H, Butler K, Yang L, Yang Z, Teng R (2012). Pharmacokinetics and tolerability of single and multiple doses of Ticagrelor in healthy Chinese subjects:an open-label, sequential, two-cohort, single-center study. Clin Drug Investig.

[10] Teng R, Butler K (2013). Effect of the CYP3A inhibitors, diltiazem and ketoconazole, on ticagrelor pharmacokinetics in healthy volunteers. J Drug Assess.

[11] Draft Guideline on Ticagrelor active ingredients.

[12] Giorgi MA, Cohen Arazi H, Gonzalez CD, Di Girolamo G (2011). Beyond efficacy:pharmacokinetic differences between clopidogrel, prasugrel and ticagrelor. Expert Opin Pharmacother.

[13] Matetzky S, Shenkman B, Guetta V, Shechter M, Beinart R, Goldenberg I (2004). Clopidogrel resistance is associated with increased risk of recurrent atherothrombotic events in patients with acute myocardial infarction. Circulation.

[14] Mu¨ller I, Besta F, Schulz C, Massberg S, Scho¨nig A, Gawaz M (2003). Prevalence of clopidogrel non-responders among patients with stable angina pectoris scheduled for elective coronary stent placement. Thromb Haemost.

[15] Tantry US, Bliden KP, Wei C (2010). First analysis of the relation between CYP2C19 genotype and pharmacodynamics in patients treated with ticagrelor versus clopidogrel. Circ Cardiovasc Genet.

